# Mutational and phenotypic spectrum of *OTOF*-related auditory neuropathy in Koreans: eliciting reciprocal interaction between bench and clinics

**DOI:** 10.1186/s12967-018-1708-z

**Published:** 2018-11-27

**Authors:** Bong Jik Kim, Jeong Hun Jang, Jin Hee Han, Hye-Rim Park, Doo Yi Oh, Seungmin Lee, Min Young Kim, Ah Reum Kim, Chung Lee, Nayoung K. D. Kim, Woong-Yang Park, Yun-Hoon Choung, Byung Yoon Choi

**Affiliations:** 1 Department of Otolaryngology-Head and Neck Surgery, Chungnam National University College of Medicine, Daejeon, 35015 Korea; 2 Department of Otorhinolaryngology-Head and Neck Surgery, Seoul National University Bundang Hospital, Seoul National University College of Medicine, 300 Gumi-dong, Bundang-gu, Seongnam, 13620 Republic of South Korea; 3 Department of Otorhinolaryngology-Head and Neck Surgery, Ajou University School of Medicine, Suwon, 16499 Korea; 4 Samsung Genome Institute, Samsung Medical Center, Seoul, 06351 Korea; 5 Department of Molecular Cell Biology, School of Medicine, Sungkyunkwan University, Seoul, 06351 Korea; 6 Sensory Organ Research Institute, Seoul National University Medical Research Center, Seoul, 03080 Korea

**Keywords:** *OTOF*, Auditory neuropathy spectrum disorder, Whole exome sequencing, DFNB9, Auditory steady-state response, Cochlear implantation

## Abstract

**Background:**

While auditory neuropathy spectrum disorder (ANSD) is a heterogeneous disorder and its management quite varies depending upon the etiology, even including self-resolution, *OTOF* is an important molecular etiology of prelingual ANSD and has emerged as an attractive target for implementation of precision medicine in terms of timing and prognosis prediction of auditory rehabilitation. However, to date, the literature is lacking in the genotype–phenotype relationship of this gene as well as efficient molecular testing strategy in the clinic in many populations and to make things more complicated in Koreans, the most prevalent variant p.Arg1939Gln among Korean ANSD children frequently evaded detection by next generation sequencing (NGS), resulting in delayed genetic diagnosis and late cochlear implantation (CI). The aims of this study are to document the mutational and phenotypic spectrum of *OTOF*-related ANSD (DFNB9) in the Korean population, further establishing genotype–phenotype correlation and proposing a set of the most commonly found *OTOF* variants to be screened first.

**Methods:**

Genetic diagnosis through the NGS-based sequencing was made on patients with ANSD in two tertiary hospitals. Genotype and phenotypes of eleven DFNB9 patients were reviewed. For data analysis, Mann–Whitney test and Fisher’s exact test were applied.

**Results:**

This study disclosed four prevalent variants in Koreans: p.Arg1939Gln with an allele frequency of 40.9%, p.Glu841Lys (13.6%), p.Leu1011Pro and p.Arg1856Trp (9.1%). Three novel variants (c.4227 + 5G > C, p.Gly1845Glu, and p.Pro1931Thr) were identified. Interestingly, a significant association of p.Arg1939Gln with worse ASSR thresholds was observed despite consistently no ABR response. Ten of 11 DFNB9 patients received CI for auditory rehabilitation, showing favorable outcomes with more rapid improvement on early-CI group (age at CI ≤ 18 mo.) than late-CI group.

**Conclusions:**

This study included the largest Korean DFNB9 cohort to date and proposed a set of the most frequent four *OTOF* variants, allowing the potential prioritization of exons during Sanger sequencing. Further, a significant association of p.Arg1939Gln homozygotes with poor residual hearing was observed. We may have to suspect p.Arg1939Gln homozygosity in cases of poor auditory thresholds in ANSD children with putative negative *OTOF* variants solely screened by NGS. Reciprocal feedback between bench and clinics regarding DFNB9 would complement each other.

## Background

Auditory neuropathy spectrum disorder (ANSD) is a hearing disorder characterized by impaired transmission of sound signals from the inner ear to the auditory nerve/brain cortex with normally-functioning outer hair cells. Clinically, ANSD is diagnosed by the presence of otoacoustic emissions (OAE) or cochlear microphonics (CM), and the absence or severe impairment of auditory brainstem response (ABR) [1]. Approximately 10% of neonates or infants diagnosed with profound hearing loss suffer from ANSD; in about 50% of them, genetic cause is suspected [2, 3]. *OTOF*, and *PJVK* in an autosomal recessive manner, *DIAPH3*in an autosomal dominant manner and a locus *AUNX1* in an X-linked recessive manner have been reported to be associated with non-syndromic ANSD [4–7]. Specifically, *OTOF*-related ANSD (DFNB9) is assumed to be caused by a pathology confined to the synapse [8–10], proximal to the postsynaptic neuron or auditory nerve, which is the site for electrical stimulation of cochlear implantation (CI). For this reason, and as evidenced by several previous studies showing good outcomes in patients receiving CI before the age of 24 months [11, 12], *OTOF*-related ANSD patients with early intervention are considered good candidates for CI. However, residual pure-tone hearing, which occurs not infrequently in ANSD children, may lead to a reluctance to perform CI earlier, unless there is a definite molecular etiologic diagnosis. Decision of timing of CI in ANSD is furthermore clinically challenging in that a subset of patients indeed manifest spontaneous hearing recovery with age [13], while ANSD patients with *OTOF* variant do not recover spontaneously.

In fact, a large size of this gene encompassing 46 exons hindered the screening of the whole gene by Sanger sequencing, causing a total dependence on next generation sequencing (NGS). Some previous studies from Korea relying on NGS, including our own, misconceived that the majority of Korean ANSD is unrelated to DFNB9 [14, 15]. Later, this misconception turned out to be mainly due to frequent capture failures and poor coverage of the last exon of the cochlear isoform of *OTOF* in the conventional panel sequencing strategy, where the predominant variant of DFNB9 in this population p.Arg1939Gln resides [16]. Resultantly, we reported that some portion of anatomically normal cochlear nerve, prelingual-onset ANSD in Koreans could be due to mutations in *OTOF* and identified that screening of p.Arg1939Gln, which is a major allele in prelingual-onset Korean ANSD, may facilitate detection of DFNB9. Interestingly p.Arg1939Gln was also recognized as a founder mutation in Japanese ANSD populations [17].

However, the overall mutational spectrum of DFNB9 in Koreans has not been fully established yet, and performing either NGS or Sanger sequencing of all the exons of *OTOF* for all ANSD children is still costly in a clinical setting. Moreover, the potential correlation between the degree of residual hearing as measured by behavioral audiometry and certain *OTOF* variants has not been clearly elucidated thus far except for a few temperature sensitive mutant alleles of this gene resulting in elevation of hearing thresholds presumably due to a synaptic dysfunction elicited by fever [18, 19]. Knowing the genotype–phenotype correlation, if any, of this large gene may significantly help to narrow down the candidate variants of *OTOF*. Since our previous report, we further recruited anatomically normal cochlear nerve, prelingual-onset ANSD subjects and confirmed again a high genetic load of *OTOF* to Korean prelingual ANSD.

Here, we disclose a mutational and phenotypic spectrum of *OTOF*-related ANSD in this Korean population and propose a set of commonly found *OTOF* variants in our population that would allow for the potential prioritization of specific *OTOF* exons during the screening process. The outcomes of CI were also addressed with respect to the timing of implantation.

## Methods

### Subjects and ethical considerations

**Fig. 1:**
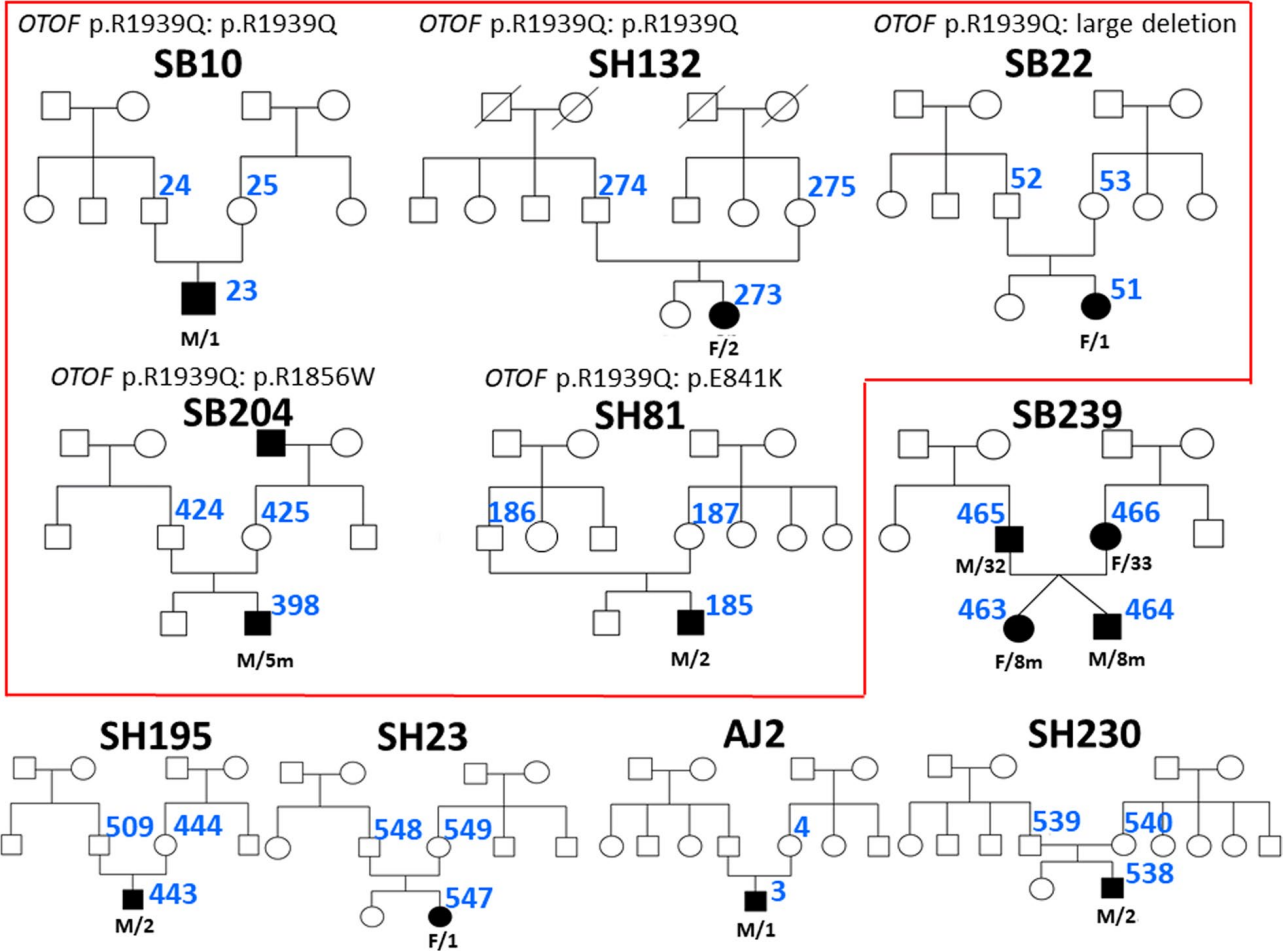
Pedigrees of DFNB9 families with molecular genetic diagnosis. Filled symbols represent hearing-impaired individuals, and clear symbols denote those with normal hearing. A diagonal line through a symbol indicates a deceased person and black arrows indicate probands. Designations below each proband represent sex/age. Previously diagnosed families are surrounded by a red border

Among the total 162 families with hereditary hearing loss ascertained from Seoul National University Hospital (SNUH) and Seoul National University Bundang Hospital (SNUBH) between June 2015 and March 2017, 78 families turned out to manifest hearing loss in an autosomal recessive or sporadic fashion. Of these 78 families, we were able to recruit five new families (SB239, SH195, SH230, SH234, and AJ2) with ANSD through rigorous audiological phenotyping [14]. Together with the five previously reported families (SB10, SB22, SB204, SH132, and SH81) [16], these additional families provided a total of ten families. Genotype and phenotype evaluations, including audiometric assessment, were made through molecular genetic diagnosis and medical chart review. Regarding SB239, two family members (SB239-465, 466) are genetically unrelated, thus they were counted distinctly in the genotypic analysis, and their two children (SB239-463, 464) were included only in the phenotype analysis. The pedigrees of participants were described (Fig. 1).

### Molecular genetic diagnosis

Molecular genetic diagnoses of 5 families with 6 probands was made through a screening kit for detection of prevalent 11 deafness-causing variants [20] and Sanger sequencing of previously reported *OTOF* variants among Koreans including p.Arg1939Gln followed by either targeted exome sequencing (TES), or whole exome sequencing (WES), as previously described [12, 21–24]. In detail, genomic DNA was extracted from the peripheral blood samples or buccal cells using standard protocols (Gentra Puregene Blood Kit, Qiagen, cat. 158389; Venlo, Limburg, 27 Netherlands). First, screening panel sequencing of 11 variants of 5 deafness genes (*GJB2*, *SLC26A4*, *CDH23*, *12S rRNA*, *TMPRSS3*) in SB239-463, 464 or Sanger sequencing of p.Arg1939Gln of *OTOF* in SH195-443, SH234-547, AJ2-3, and SH230-538 was performed based on the prevalence of deaf genes in the Korean population. If a potential candidate variant(s) of *OTOF* was detected, then detailed Sanger sequencing of *OTOF* was performed to find another candidate variant causing a recessive form of DFNB9 (SH234). If no variant was detected in the screening, then TES129 (SH195, SH230) or WES (SB239, AJ2) followed [22]. Candidate variants were narrowed down filtered through a bioinformatics analysis of the data and selected variants were confirmed by Sanger sequencing. The filtered variants were evaluated for pathogenicity using various in silico prediction software as previously described [14, 25].

### Audiometric evaluation

Pure-tone audiometry, ABR, OAE, including transient-evoked OAE (TEOAE) or distortion product OAE (DPOAE), and/or Auditory Steady-State Response (ASSR) were performed to assess the hearing status. Because it was difficult to acquire behavioral audiometry from children under 3 years old, ASSR was performed to infer the auditory threshold; ASSR threshold of 500 Hz was used as a reliable factor to correlate with the auditory threshold [26]. Hearing level calculated by the four-tone average (0.5, 1, 2, and 4 kHz) was labeled as mild (26–40 dB), moderate (41–70 dB), severe (71–90 dB), or profound (> 90 dB) and a difference between the left and right ear air conduction threshold > 20 dB at least two frequencies out of 0.5, 1, and 2 kHz was indicated as asymmetric. Speech perception performance was assessed with modified categories of auditory perception (CAP) scores, which evaluates the auditory receptive ability with a nonlinear, hierarchical scale [27], before and after CI in case of CI recipients.

### Statistical analysis

The data are expressed as the mean ± SE. Mann–Whitney test was used to compare the means of postoperative CAP scores between early-CI and late-CI groups at each time point, and the mean ASSR threshold at 500 Hz between patients with homozygous p.Arg1939Gln and non-carriers. Fisher’s exact test was used to determine the association of frequency of p.Arg1939Gln allele in accordance with the timing of CI (early and late-CI group). Statistical analyses were performed and graphs were plotted using GraphPad prism 5.0 software (GraphPad Software, La Jolla, CA, USA). A *p* value of < 0.05 was considered statistically significant.

## Results

### Mutational spectrum of DFNB9 in Koreans

We analyzed six unrelated DFNB9 subjects, making the total number of unrelated DFNB9 subjects to 11 (Table 1). The molecular genetic approach adopted in our cohort identified eight different variants and one large genomic deletion of the *OTOF* gene, of which, three variants segregating in three families were novel (Table 1). Specifically, the inhibitory effect of the c.4227 + 5G > C on the splice donor site was suggested by splice site variant prediction tools, which strongly suggested that the c.4227 + 5G > C splicing variant significantly impairs the canonical splice donor site of exon 34.

**Table 1:** Mutational spectrum of 11 subjects from 10 families with DFNB9

Family ID	Variant (*OTOF*) NM_001287489 NP_001274418	State	Prediction algorithm			Conservation score		MAF		Classification of pathogenic variants [31]	References
			Mutation taster	PolyPhen-2	SIFT	PhyloP	GERP++	Global MAF	KRGDB (n = 1722)		
SB10-23	c.5816G > A: p.Arg1939Gln rs201326023	Hom	DC	PD	D	2.261	2.28	T = 0.00003/1 (ExAC) T = 0.0002/1 (1000 Genomes)	T = 0.001452/5	Pathogenic (PS4, PM2, PM3 PP1, PP3, PP4)	[31]
SH132-273	c.5816G > A: p.Arg1939Gln rs201326023	Hom	DC	PD	D	2.261	2.28	T = 0.00003/1 (ExAC) T = 0.0002/1 (1000 Genomes)	T = 0.001452/5	Pathogenic	[31]
SB22-51	c.5816G > A: p.Arg1939Gln rs201326023	Het	DC	PD	D	2.261	2.28	T = 0.00003/1 (ExAC) T = 0.0002/1 (1000 Genomes)	T = 0.001452/5	Pathogenic	[31]
	Large genomic deletion Chr2:26710657 ~ 26706557	Het	NA	NA	NA	NA	NA			Pathogenic (PVS1, PP1, PP4)	[16]
SB204-398	c.5816G > A: p.Arg1939Gln rs201326023	Het	DC	PD	D	2.261	2.28	T = 0.00003/1 (ExAC) T = 0.0002/1 (1000 Genomes)	T = 0.001452/5	Pathogenic	[31]
	c.5566C > T: p.Arg1856Trp rs368155547	Het	DC	PD	D	2.963	2.84	A = 0.00004/5 (ExAC) A = 0.00008/1 (GO-ESP)	A = 0.000871/3	Pathogenic (PS4, PM2, PM3 PP1, PP3, PP4)	[10]
SH81-185	c.5816G > A: p.Arg1939Gln rs201326023	Het	DC	PD	D	2.261	2.28	T = 0.00003/1 (ExAC) T = 0.0002/1 (1000 Genomes)	T = 0.001452/5	Pathogenic	[31]
	c.2521G > A: p.Glu841Lys rs772729658	Het	DC	PD	D	5.523	5	T = 0.00003/3 (ExAC)	ND	Pathogenic (PS4, PM2, PM3 PP1, PP3, PP4)	[16]
SB239-465	c.5816G > A: p.Arg1939Gln rs201326023	Het	DC	PD	D	2.261	2.28	T = 0.00003/1 (ExAC) T = 0.0002/1 (1000 Genomes)	T = 0.001452/5	Pathogenic	[31]
	c.3032T > C: p.Leu1011Pro rs80356596	Het	DC	PD	D	5.012	4.64	ND	ND	Pathogenic (PS4, PM2, PM3 PP1, PP3, PP4)	[32]
SB239-466	^a^c.5791C > A: p.Pro1931Thr rs537706054	Het	DC	PD	D	5.867	5.22	T = 0.000008/1 (ExAC)	ND	Pathogenic (PM2, PM3, PP1, PP3, PP4)	This study
	c.2521G > A: p.Glu841Lys rs772729658	Het	DC	PD	D	5.523	5	T = 0.00003/3 (ExAC)	ND	Pathogenic	[16]
SH195-443	c.3192C > G: p.Tyr1064Ter rs766819324	Hom	DC	NA	NA	1.937	2.78	C = 0.000008/1 (ExAC)	C = 0.00029/1	Pathogenic (PVS1, PM2, PP1, PP3, PP4)	[15]
SH234-547	c.5816G > A: p.Arg1939Gln rs201326023	Het	DC	PD	D	2.261	2.28	T = 0.00003/1 (ExAC) T = 0.0002/1 (1000 Genomes)	T = 0.001452/5	Pathogenic	[31]
	c.5566C > T: p.Arg1856Trp rs368155547	Het	DC	PD	D	2.963	2.84	A = 0.00004/5 (ExAC) A = 0.00008/1 (GO-ESP)	A = 0.000871/3	Pathogenic	[10]
AJ2-3	^a^c.5534G > A: p.Gly1845Glu dbSNP ID:ND	Het	DC	PD	D	5.739	4.97	ND	ND	Pathogenic (PM2, PM3 PP1, PP3, PP4)	This study
	c.3032T > C: p.Leu1011Pro rs80356596	Het	DC	PD	D	5.012	4.64	ND	ND	Pathogenic	[32]
SH230-538	c.2521G > A: p.Glu841Lys rs772729658	Het	DC	PD	D	5.523	5	T = 0.00003/3 (ExAC)	ND	Pathogenic	[16]
	^a^c.4227 + 5G > C rs571671530	Het	DC	NA	NA	1.616	3.95	G = 0.00006/7 (ExAC) G = 0.0002/1 (1000 Genomes)	ND	Likely pathogenic (PM2, PM3 PP1, PP3, PP4)	This study

Splice site variant prediction tools by ESEfinder, NNSplice, and NetGene2: splice site broken. Normal score (10.34940) and mutant score (6.54650) by ESEfinder, Normal score (0.99) and mutant score (0.50) by NNSplice, Normal score (0.997) and mutant score (0.685) by NetGene2

In silico prediction Algorithm: Polyphen-2 (http://genetics.bwh.harvard.edu/pph2/index.shtml); SIFT (http://sift.jcvi.org/www/SIFT_chr_coords_submit.html) or SIFT-indels2 (http://sift.bii.a-star.edu.sg/www/SIFT_indels2.html)

Conservation tools: GERP++ score in the UCSC Genome Browser (http://genome-asia.ucsc.edu/); PhyloP score from the Mutation Taster (http://www.mutationtaster.org/)

Splice site prediction tools: ESEfinder (http://rulai.cshl.edu/cgi-bin/tools/ESE3/esefinder.cgi?process=home); NNSplice (http://www.fruitfly.org/seq_tools/splice.html); NetGene2 (http://www.cbs.dtu.dk/services/NetGene2/)

ExAC, Exome Aggregation Consortium (http://exac.broadinstitute.org/)

1000 Genomes (https://www.ncbi.nlm.nih.gov/variation/tools/1000genomes/)

KRGDB, Korean Reference Genome DB (http://152.99.75.168/KRGDB/)

Het, heterozygote mutant; Hom, homozygote mutant; DC, disease causing; PD, probably damaging; D, damaging; ND, not detected; NA, not applicable

^a^Novel variant

**Fig. 2:**
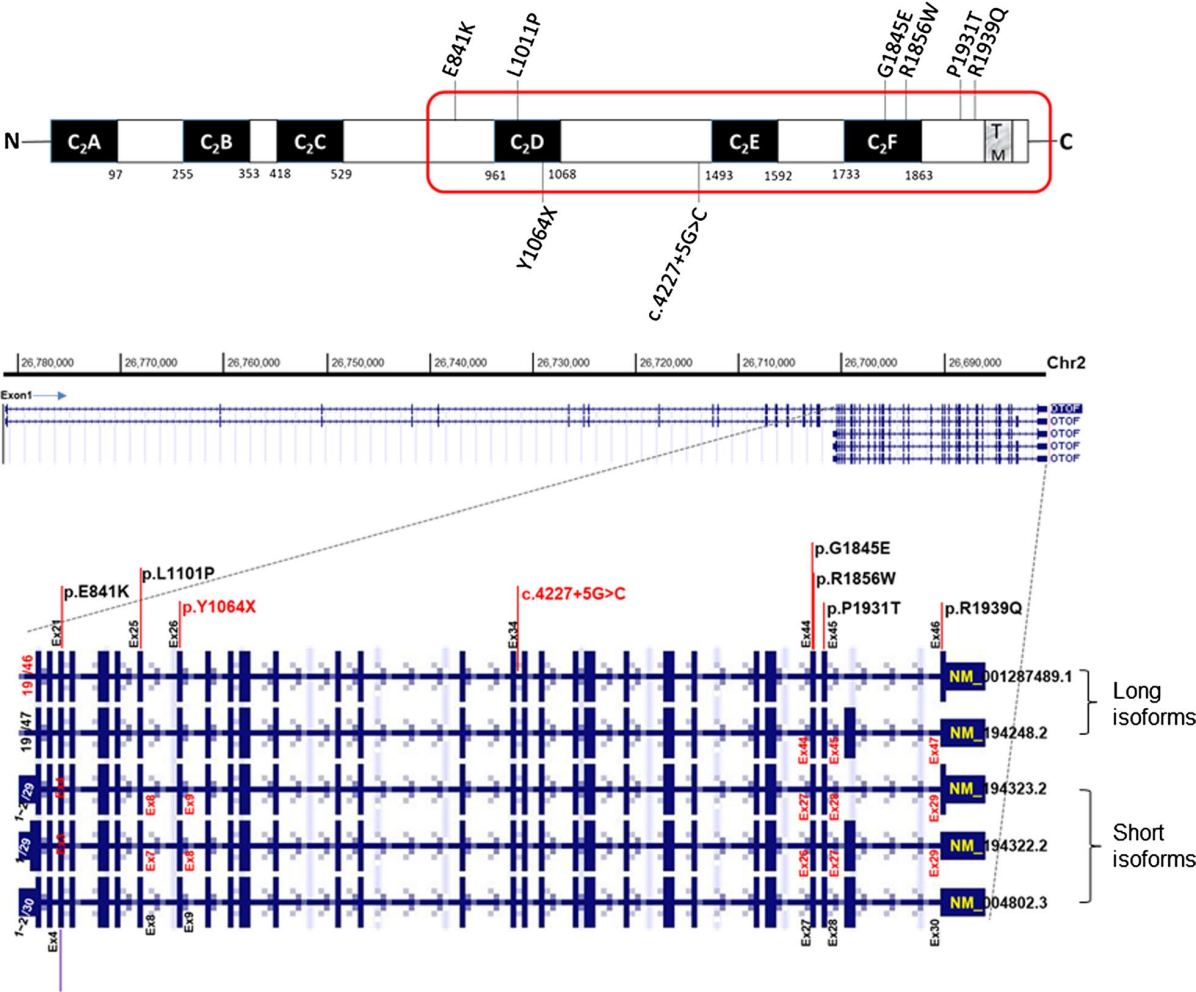
Protein domain structures of otoferlin with pathogenic variants in our cohort. Upper panel: Otoferlin contains six C_2_ domains (C_2_A–C_2_F). Missense variants are displayed (top) on the domain structure, and nonsense and splice site variants are located below the domain structure. A red bordered area contains three C_2_ domains encoded by short isoform of *OTOF*. TM: transmembrane domain. Lower panel: Exon–Intron structure of various *OTOF* isoforms (NM_001287489, NM_194248, NM_194323, NM_194322, NM_004802) locating each variant detected in the study (Captured from UCSC genome browser (https://genome.ucsc.edu/))

Taken together with the data from the previous study, six different kinds of missense variants, one nonsense variant, one splice site variant, and one large deletion were found in our cohort of 11 DFNB9, estimating the contribution of *OTOF* etiology to be 90.9% (10 DFNB9 pedigrees/11 ANSD families) among confirmed prelingual ANSD patients in our Korean deaf cohorts. Regarding the frequency of *OTOF* variants, p.Arg1939Gln was most commonly identified among the 22 alleles in 11 DFNB9 subjects, with a frequency of 40.9% (9/22). The variant p.Glu841Lys was the second most frequent (13.6%, 3/22), followed by two variants, p.Leu1011Pro and p.Arg1856Trp, which accounted for 9.1% (2/22) each. The total contribution of these four major alleles was 72.7% among all ANSD patients with *OTOF* variants. The detected variants in the *OTOF* gene were distributed ranging from exon 21–46, clustered around C_2_D, C_2_E, and C_2_F domains of otoferlin (Fig. 2).

### Phenotypic spectrum of DFNB9 in Koreans

Temporal bone CT or internal auditory canal MRI clearly revealed no anatomical abnormalities of the cochlear nerve in the 11 probands. Regarding newborn hearing screening (NBHS), one patient (SH230-538) passed the NBHS even with significant hearing loss because only OAE was performed; the other patient (SH132-273) did not undergo NBHS. All patients, except these two, failed NBHS, facilitating an early detection of hearing loss and leading to a molecular genetic diagnosis of DFNB9, and no fever-induced hearing aggravation was observed.

**Fig. 3:**
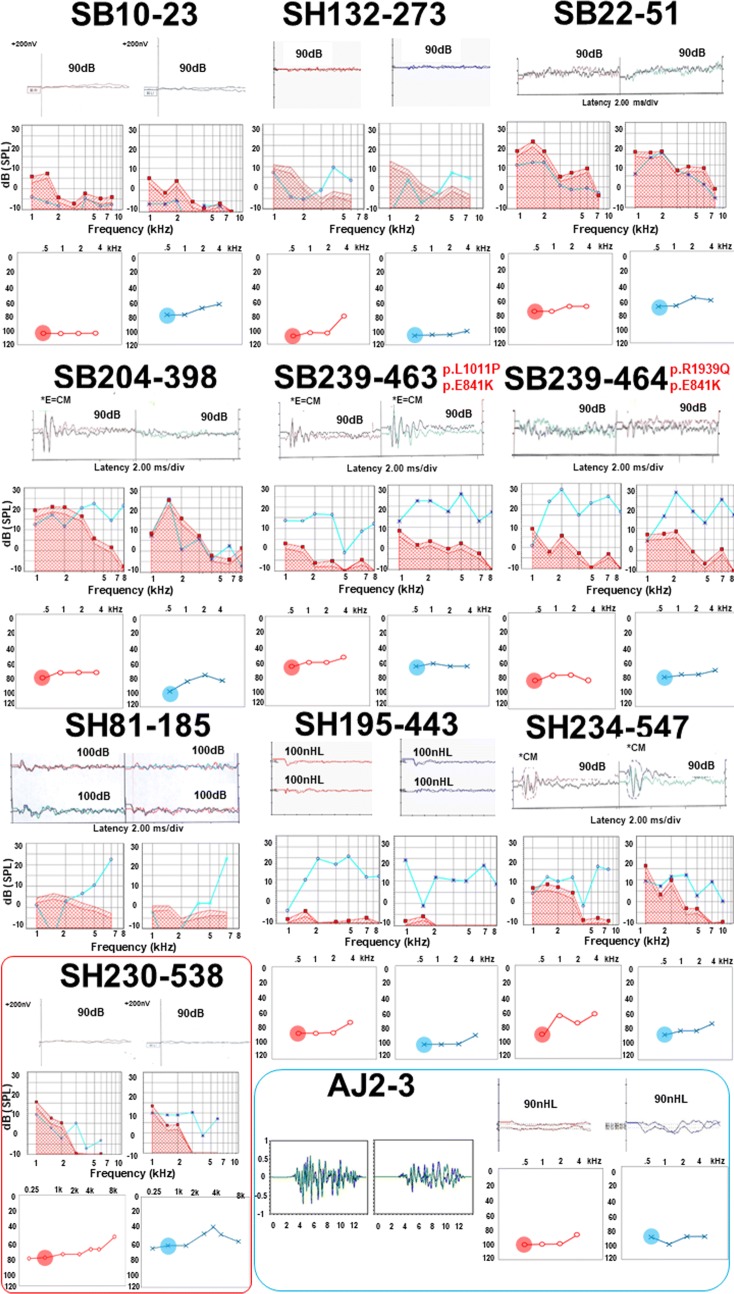
Audiological phenotypes of affected members in ten families with DFNB9. ABR, OAE, and ASSR results are described in order in each patient. Each number on each side of ABR (right in red and left in blue) represents the stimulus intensity calibrated in dB or nHL, which showed no response in all patients. Auditory thresholds of ASSR at 500 Hz are circled in red and blue, each for right and left sides, respectively

Detailed audiological results of eleven patients are described in Fig. 3 and Table 2. The results of ABR did not show any noticeable responses on both sides, and OAEs indicated the presence of response in accordance with the definition of ANSD. On the contrary, ASSR recordings demonstrated various results, including asymmetric or low-frequency hearing loss configuration. In detail, one patient (SB10-23) showed asymmetric hearing loss, and another patient (SH230-538) had characteristic low-frequency hearing loss on both sides. The severity ranged mostly from severe to profound only with some exceptions (SB22-51, SB239-463, SH230-538) based on ASSR results. All patients who had no response with ABR test, presented measurable ASSR thresholds, showing the possibility of providing more detailed hearing level and configuration.

**Table 2:** Phenotypic spectrum of 11 subjects (including SB239-463, 464) from 10 families with DFNB9

Family ID	Genotype variant (*OTOF*) NM_001287489 NP_001274418	Age at first CI (month)	Laterality	Pre-CI assessment				Post-CI CAP score				
				ABR (dB)	OAE	ASSR at 500 Hz (dB)	CAP score	3 m	6 m	12 m	24 m	36 m
SB10-23	c.5816G > A: p.Arg1939Gln homozygous state	21	Bilateral, Sequential	B) NR	L) response (+)	R) 105 L) 80	0	1	1	4	6	7
SH132-273	c.5816G > A: p.Arg1939Gln homozygous state	22	Bilateral, Sequential	B) NR	B) response (+)	R) 110 L) 105	0	2	3	5	7	
SB22-51	c.5816G > A: p.Arg1939Gln Large genomic deletion (Chr2:26710657 ~ 26706557)	26	Bilateral, Sequential	B) NR	L) response (+)	R) 75 L) 70	0	1		5	5	7
SB204-398	c.5816G > A: p.Arg1939Gln c.5566C > T: p.Arg1856Trp	13	Bilateral, Simulatneous	B) NR	B) response (+)	R) 80 L) 100	0	2	4	4	5	
SH81-185	c.5816G > A: p.Arg1939Gln c.2521G > A: p.Glu841Lys	25	Bilateral, Sequential	B) NR	B) response (+)	NA	0	1	1	4	4	
SB239-463	c.2521G > A: p.Glu841Lys c.3032T > C: p.Leu1011Pro	14	Bilateral, Simulatneous	B) NR	B) response (+)	R) 65 L) 65	1	3	5	5		
SB239-464	c.5816G > A: p.Arg1939Gln c.2521G > A: p.Glu841Lys	14	Bilateral, Simulatneous	B) NR	B) response (+)	R) 80 L) 80	0	2	4	5		
SH195-443	c.3192C > G: p.Tyr1064Ter homozygous state	24	Bilateral, Simulatneous	B) NR	B) response (+)	R) 85 L) 100	1	3	FU loss			
SH234-547	c.5816G > A: p.Arg1939Gln c.5566C > T: p.Arg1856Trp	15	Bilateral, Simulatneous	B) NR	B) response (+)	R) 90 L) 85	0	3				
AJ2-3	c.5534G > A: p.Gly1845Glu c.3032T > C: p.Leu1011Pro	18	Bilateral, Sequential	B) NR	B) response (+)	R) 100 L) 90	0	4	4			
SH230-538	c.4227 + 5G > C c.2521G > A: p.Glu841Lys	Scheduled	NA	B) NR	B) response (+)	R) 75 L) 60	0					

NR, no response; NA, not applicable

We observed a slight genotype–phenotype correlation in one family (SB239), where even the intra-familial phenotypic variations were found. In SB239, there are four family members having DFNB9 (parents are biological parents and genetically unrelated). In detail, SB239-463 (daughter: compound heterozygous state carrying p.Glu841Lys and p.Leu1011Pro) showed better pre-CI CAP score and ASSR hearing thresholds at 500 Hz than her dizygotic twin brother SB239-464 carrying p.Arg1939Gln along with p.Glu841Lys (Fig. 3). Likewise, of their two parents who had received CIs during adulthood, the father (SB239-465) carrying p.Arg1939Gln did not improve in listening skills with auditory rehabilitation, while the mother (SB239-466) without p.Arg1939Gln demonstrated great improvement, showing a stark contrast with her husband under similar clinical settings.

Given that only ASSR thresholds at 500 Hz were reported to correlate with behavioral thresholds from ANSD subjects [26], we specifically pursued the issue of genotype–phenotype correlation using the thresholds at 500 Hz. ASSR thresholds at 500 Hz from p.Arg1939Gln homozygotes (n = 2) were higher compared with those from the p.Arg1939Gln non-carriers (*p *= 0.033) (n = 4), and this significant phenotypic difference was also reproduced for a comparison between p.Arg1939Gln homozygotes and all the others (n = 8) including p.Arg1939Gln single heterozygotes (*p *= 0.022) although the sample size was small to draw a firm conclusion.

**Fig. 4:**
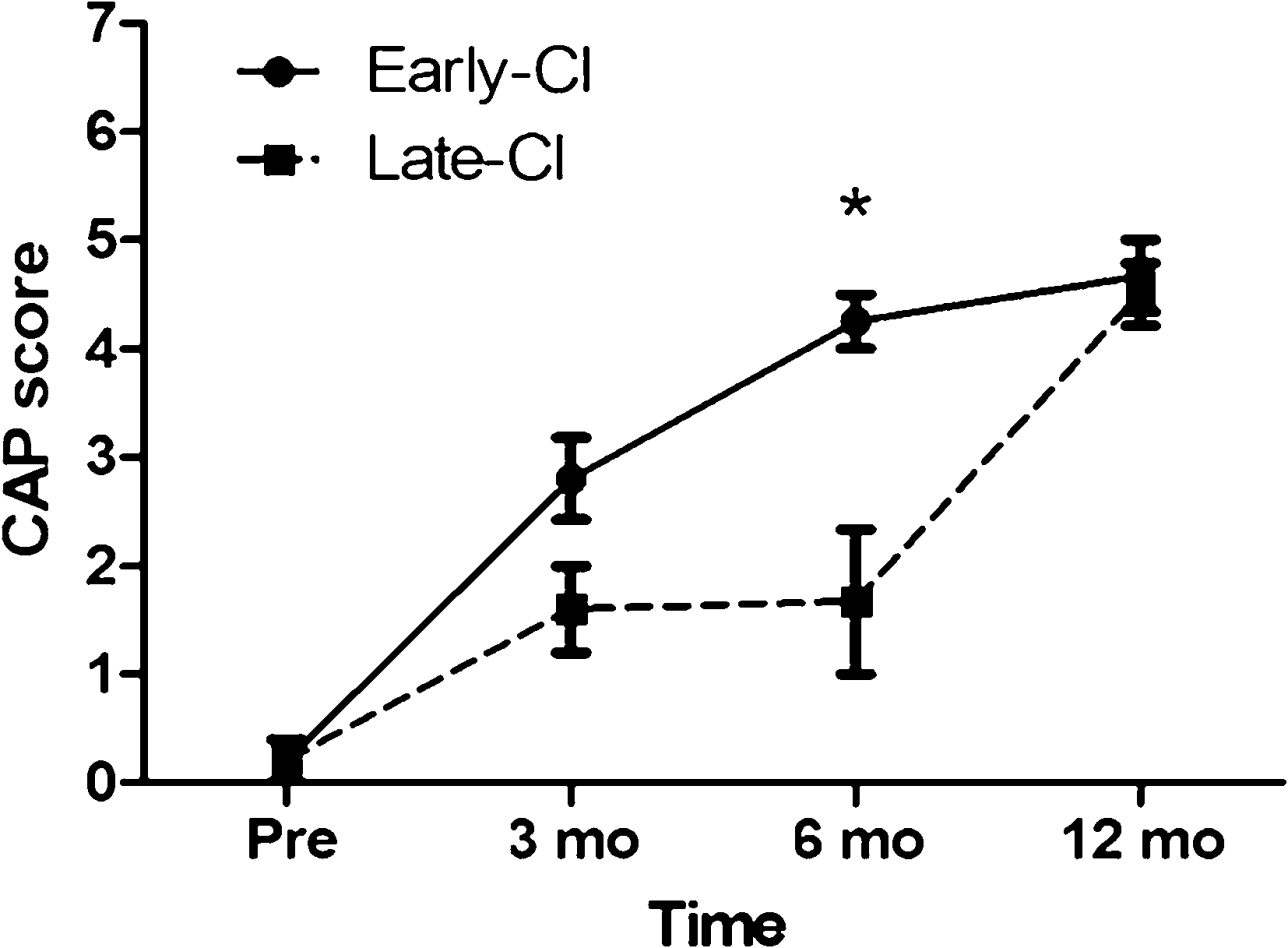
Average CAP scores of CI recipients. Audiologic performances of the two subgroups (early-CI and late-CI) at pre- and post-CI were analyzed longitudinally (*p *= 0.04 and 0.08 at post-CI 3 mo and 6 mo respectively). CAP is composed of a nonlinear, hierarchical scale with eight categories: (0) Displays no awareness of environmental sounds; (1) awareness of environmental sounds; (2) responds to speech sounds; (3) recognizes environmental sounds; (4) discriminates at least two speech sounds; (5) understands common phrases without lip-reading; (6) understands conversation without lip-reading with a familiar talker; (7) can use telephone with a familiar talker

With respect to auditory rehabilitation in our cohort, ten out of eleven patients underwent bilateral CI, either sequentially or simultaneously, and one patient (SH230-538) is scheduled to have one (Table 2). Based on the CAP score, our cohort showed comparable postoperative auditory outcomes with those observed in cochlear implantees with other etiologies [12]. For further analysis, CI recipients were divided into two groups: early (age at CI ≤ 18mo) and late-CI groups (age at CI > 18mo) with potential waiting time for CI in ANSD patients in a clinical setting considered. Longitudinal changes in the auditory performance (CAP scores) were compared between the early (n = 5) and late-CI groups (n = 5) (Fig. 4). The early-CI group showed better outcomes than the late-CI group at post-CI 6mo (*p *= 0.04), but the difference became minimal by post-CI 12 mo. The p.Arg1939Gln allele tended to be more frequently detected in the late-CI group (60%) than in the early-CI group (30%), but without statistical significance (*p *= 0.37).

## Discussion

In this study, which included the largest Korean cohort to date, a strong genetic etiology of ANSD in Korean pediatric deaf population was confirmed again, with alterations of *OTOF* explaining 91% of ANSD patients and we were able to come up with a set of four recurring variants that allow screening of 72.7% of the total *OTOF* variants detected in Korean ANSD children. Moreover, our study identified three novel *OTOF* variants, which comprised of two missense variants and one splice site variant. Interestingly, all variants detected in our cohort were distributed in relatively limited parts of the *OTOF* gene, ranging between exons 21 and 46. It is worth noting that all of the *OTOF* variants detected in Korean DFNB9 subjects are confined to the common regions shared by both short and long isoforms, affecting both of the two isoforms. We could not detect the pathogenic variants that were present exclusively in the long isoform-specific sequences (from exon 1 through exon 20) from our cohorts. However, in other ethnic populations, variants residing in the sequences specific to the long isoforms are present, clearly establishing the requirement of long isoforms of otoferlin for hearing in the literature [10]. As such, the confined characteristic of Korean *OTOF* variants to the region shared by both long and short isoforms is contrasted with the dispersed characteristic of Pakistani DFNB9 subjects, in which several private variants are scattered all across the entire long isoforms [10]. It is worth mentioning that a predominantly recurring variant, such as p.Arg1939Gln in Koreans, does not exist in the Pakistani population. Preponderance of a small number of variants only in the limited region clearly offers an advantageous condition from the perspectives of mutation screening strategy, which should also consider the future discovery of other pathogenic *OTOF* variants in more representative samples of Korean ANSD patients.

In terms of auditory phenotypes, ASSR showed diverse configurations and thresholds in spite of no response in the ABR test throughout our entire prelingual ANSD cohort. The variable degree of reduced neurotransmitter release in each variant or each patient might be a cause of various ASSR results [8]. However, these ASSR results need to be interpreted cautiously given the characteristics of ASSR. ASSR has its merits for evaluating hearing in children with hearing threshold beyond the limit of ABR, and it could also measure more frequency-specific hearing threshold than ABR [28]. The variance in ASSR results compared with the consistency of no response in ABR recordings may suggest hidden residual hearing or frequency-specific hearing threshold not evaluated by ABR. However, its reliability in evaluating middle-frequency hearing is relatively low; hence, ASSR alone may be insufficient, requiring a combination with other hearing assessment tools, including ABR. In this study, the ASSR threshold only at 500 Hz was used to infer the behavioral threshold [26]. This prompted us to extend the analysis to include all ASSR thresholds at 500 Hz of our DFNB9 cohort and evaluate whether there is any auditory phenotype-genotype correlation among DFNB9 subjects using this parameter. The elucidation of this correlation, if any, might in turn help to suspect certain *OTOF* variants, which may facilitate molecular diagnosis of DFNB9 and eventually ensure early CI for better outcomes. Inspired by the intra-familial phenotypic difference from family 239, we speculated that there might be some differences in the pathogenic potential between those with and without p.Arg1939Gln that causes this kind of variations. Previously, more severe phenotype with p.Arg1939Gln than other non-truncating variants was suggested, which could support the possible genotype–phenotype correlation that contributed to the intra-familial phenotypic differences [17]. In our study, significantly worse ASSR thresholds at 500 Hz of patients with homozygous p.Arg1939Gln compared with those with non-p.Arg1939Gln homozygotes and those with non-p.Arg1939Gln carriers were observed although the interpretation should be made cautiously due to the small sample size. However, at least we could say that in case of ANSD subjects with null residual hearing, we may have to think about the p. Arg1939Gln homozygous phenotype, which frequently escapes capture in NGS. The exclusion of DFNB9 by just relying on the NGS results may be risky in these cases.

Interestingly, the p.Arg1939Gln allele tended to be more frequently detected in the late-CI group (60%) than in early-CI group (30%). This tendency might have been partly due to the frequent capture failures through NGS in screening the last exon carrying p. Arg1939Gln although no statistical inferences can be made due to the small sample size. Many surgeons tend to delay CI in ANSD children, at least until the child is 18 months old, unless they are given a definite molecular diagnosis of DFNB9. If the molecular genetic diagnosis is made earlier, it may be possible to perform CI sooner, rather than later. Given this, the elucidation of a set of frequently occurring *OTOF* variants that can be easily screened by Sanger sequencing in Koreans may significantly contribute to earlier CI in DFNB9 children, which comprised a majority of ANSD children in Koreans.

In line with the previous report [11], the CI outcomes of our DFNB9 cohorts also demonstrated favorable results, with small variance, according to the timing of implantation (early vs. late) although small sample size undermined the conclusion. This phenomenon might be partly due to the fact that the late-CI group also received CI around 24 months, which was still regarded as the acceptable period for proper auditory development [29].

Then, why is the p.Arg1939Gln allele associated with poor ASSR thresholds? No possible explanations were proposed yet for this frequent allele as well as other DFNB9 alleles. *OTOF* encodes the multi-C_2_ domain protein otoferlin, whose alterations are associated with human prelingual deafness DFNB9 [4]. Otoferlin was reported to play a key role in synaptic sound encoding by promoting exocytosis in the inner hair cells and priming the vesicle in the presynaptic area [30]. There have been previous reports about temperature sensitive elevation of hearing thresholds in humans presumably caused by a synaptic dysfunction elicited by fever [18, 19]. In a recent study comparing mice to humans, mice with homozygous *Otof* mutation (*Otof*^*I515T/I515T*^) were less susceptible to heat than humans with the same variants, and temperature sensitivity of the phenotype observed in humans was assumed to be related with alterations in arginine-rich RXR motifs, which were located in positions 1244–1263 only in the human reference sequences (NP_001274418), and not in mice, causing heat instability of otoferlin [30]. According to the article, there seems to be heat sensitivity within otoferlin itself, which could worsen with mutations that weaken intermolecular interactions—like the Ile515Thr mutation that was predicted to exchange a hydrophobic residue in the hydrophobic core of the C_2_C domain against a more hydrophilic residue. In our study, no variant seems to be associated with such a RXR motif described previously [30], and no fever-induced deafness was observed. However, temperature sensitivity observed in some DFNB9 needs further evaluation and analysis and an in vivo or in vitro study using molecular biology tools will help us elucidating the mechanism underlying the genotype–phenotype correlation in the near future.

## Conclusions

Here, we report four major variants p.Arg1939Gln, p.Glu841Lys, p.Leu1011Pro, and p.Arg1856Trp—responsible for 73% of our Korean DFNB9 cohort. We believe that these variants can be implemented into the genetic screening of ANSD patients in Korea. Sanger sequencing of exons 21 through 46 could also be considered as a next tier of mutation screening in Korean patients with prelingual ANSD, especially when there is a lack of conclusive results of *OTOF* genotypes from screening the four major *OTOF* variants and/or when NGS technologies are not available. Early genetic diagnosis of *OTOF*-related ANSD could allow for greater accuracy in counseling of auditory rehabilitation and sooner clinical decision to lead to better auditory outcomes. Our study may suggest that encountering poorer ASSR thresholds in potential DFNB9 candidates might be more preferentially related to p.Arg1939Gln homozygotes, of which genotype specific to East Asians have been frequently undiagnosed solely by NGS technologies. The intimate reciprocal interaction and feedback between genotypes and auditory phenotype related to DFNB9 may complement the drawbacks of the existing molecular diagnostic tests that target DFNB9, which in turn, would result in earlier implantation and better CI outcomes.

## Abbreviations

ANSD: auditory neuropathy spectrum disorder
NGS: next generation sequencing
CI: cochlear implantation
OAE: otoacoustic emissions
CM: cochlear microphonics
ABR: auditory brainstem response
TES: targeted exome sequencing
WES: whole exome sequencing
PTA: pure-tone audiometry
ASSR: auditory steady-state response
CAP: categories of auditory perception
NBHS: newborn hearing screening
